# Harnessing the potential of the NALT and BALT as targets for immunomodulation using engineering strategies to enhance mucosal uptake

**DOI:** 10.3389/fimmu.2024.1419527

**Published:** 2024-09-02

**Authors:** Madison L. Seefeld, Erin L. Templeton, Justin M. Lehtinen, Noah Sinclair, Daman Yadav, Brittany L. Hartwell

**Affiliations:** ^1^ Department of Biomedical Engineering, University of Minnesota, Minneapolis, MN, United States; ^2^ Center for Immunology, University of Minnesota, Minneapolis, MN, United States

**Keywords:** nasal associated lymphoid tissue (NALT), bronchus associated lymphoid tissue (BALT), secretory IgA (SIgA), mucosal vaccine, antigen specific immunotherapy (ASIT), drug delivery, adaptive immunity, germinal center (GC)

## Abstract

Mucosal barrier tissues and their mucosal associated lymphoid tissues (MALT) are attractive targets for vaccines and immunotherapies due to their roles in both priming and regulating adaptive immune responses. The upper and lower respiratory mucosae, in particular, possess unique properties: a vast surface area responsible for frontline protection against inhaled pathogens but also simultaneous tight regulation of homeostasis against a continuous backdrop of non-pathogenic antigen exposure. Within the upper and lower respiratory tract, the nasal and bronchial associated lymphoid tissues (NALT and BALT, respectively) are key sites where antigen-specific immune responses are orchestrated against inhaled antigens, serving as critical training grounds for adaptive immunity. Many infectious diseases are transmitted via respiratory mucosal sites, highlighting the need for vaccines that can activate resident frontline immune protection in these tissues to block infection. While traditional parenteral vaccines that are injected tend to elicit weak immunity in mucosal tissues, mucosal vaccines (i.e., that are administered intranasally) are capable of eliciting both systemic and mucosal immunity in tandem by initiating immune responses in the MALT. In contrast, administering antigen to mucosal tissues in the absence of adjuvant or costimulatory signals can instead induce antigen-specific tolerance by exploiting regulatory mechanisms inherent to MALT, holding potential for mucosal immunotherapies to treat autoimmunity. Yet despite being well motivated by mucosal biology, development of both mucosal subunit vaccines and immunotherapies has historically been plagued by poor drug delivery across mucosal barriers, resulting in weak efficacy, short-lived responses, and to-date a lack of clinical translation. Development of engineering strategies that can overcome barriers to mucosal delivery are thus critical for translation of mucosal subunit vaccines and immunotherapies. This review covers engineering strategies to enhance mucosal uptake via active targeting and passive transport mechanisms, with a parallel focus on mechanisms of immune activation and regulation in the respiratory mucosa. By combining engineering strategies for enhanced mucosal delivery with a better understanding of immune mechanisms in the NALT and BALT, we hope to illustrate the potential of these mucosal sites as targets for immunomodulation.

## Introduction

Mucosal barrier tissues and mucosa-associated lymphoid tissues (MALTs) are positioned to both prime immunogenic responses against pathogens and strictly regulate tolerance against continuous background exposure to non-pathogenic antigens. The duality of mucosal tissues to either activate or suppress an immune response highlights their unique potential as a target for both immunogenic vaccines and tolerogenic immunotherapies, respectively. The respiratory tract, in particular, possesses unique immunological properties: a vast surface area responsible for frontline protection against respiratory pathogens and tight regulation of homeostasis through inherent tolerogenic mechanisms. Yet despite the promise of these tissues as a target for immunomodulation, few mucosal vaccines and immunotherapies have successfully translated to clinical use.

As of 2020, lower respiratory infections were the fourth leading cause of death worldwide. SARS-CoV-2 alone has resulted in nearly 800 million cases and over 7 million deaths globally at the time of this writing (source: World Health Organization). While current SARS-CoV-2 intramuscular vaccines have saved countless lives, they do not prevent infection or transmission as they elicit weak mucosal immunity in the respiratory tract (1). To combat persisting and emerging infectious threats from SARS-CoV-2 and other mucosally transmitted pathogens, immunization strategies are needed that elicit immune protection at mucosal portals of entry to better block infection and transmission. Mucosal vaccines that target the respiratory tract (i.e., those administered intranasally or intratracheally) provide a number of immunological advantages over traditional parenteral vaccines: they more closely mimic the route of natural infection of respiratory pathogens and prime immune responses in MALT to generate protective immunity at barrier tissues as a ‘frontline’ of defense (2, 3). Additionally, mucosal vaccines provide practical advantages over parenteral vaccines, namely the potential for greater reach and immunization rates since needle-free administration typically does not require personnel with medical training, yields higher patient compliance, and avoids risks of blood-borne pathogen spread from needle contamination (2).

Mucosal vaccination is known to be effective for promoting immunity at barrier tissues through the initiation of immune responses in underlying MALT (3). Antigen delivery to MALT can drive programming of mucosa-specific lymphocyte function and mucosal tissue homing (4) based on the division of the mucosal immune system into *inductive sites* and *effector sites* (5). Adaptive immune responses are primed at *inductive sites* (containing MALT), resulting in the generation of antigen-specific lymphocytes and plasma cells that acquire chemokine receptors for homing to local or distal *effector sites*. Nasal-associated lymphoid tissue (NALT) is the MALT and mucosal inductive site of the upper respiratory tract, considered the ‘immune sentinel’ of the respiratory tract as it primes local protective immunity (5). Bronchus-associated lymphoid tissue (BALT) is the MALT of the lower respiratory tract; for many respiratory threats it acts as the first line of defense for children who are not yet fully immunocompetent and is critically important for the establishment of lifelong immunity. Cervical lymph nodes (LNs), as proximal LNs that drain the head and neck including the nose and NALT, complement the NALT and BALT as an additional site for priming mucosal cellular and humoral immune responses following respiratory infection and immunization (6, 7). Activated memory lymphocytes and plasma cells primed in respiratory MALT establish tissue residence in the respiratory tract and remain stable long term, providing long-lived protection against respiratory infection. For example, cytotoxic CD8+ T cells that have been primed in mouse NALT and cervical LNs following intranasal immunization can become local tissue-resident memory T cells (Trm cells) that play an important role in halting transmission of virus from the upper to lower respiratory tract (8). Furthermore, lymphocytes and plasma cells primed in respiratory tissues can home *beyond* the upper and lower respiratory tract to the genitourinary mucosa, salivary gland, and bone marrow (9–11). Due to this interconnectivity (termed ‘common mucosal immunity’) and the potential to establish resident ‘gatekeepers’ across multiple mucosal tissues, vaccination by the respiratory route is a promising strategy for respiratory pathogens as well as pathogens transmitted through the genitourinary tract (12, 13).

Certain mucosal sites such as the gut and lungs are inherently predisposed to tolerance upon antigen exposure given the abundance of non-pathogenic antigens regularly encountered at these sites (i.e., food antigens, respiratory allergens, normal flora, etc.) (14, 15). This presents a challenge for mucosal vaccines as they must overcome inherent tolerance mechanisms to stimulate a robust and long-lasting immunogenic response, often achieved through the inclusion of strong adjuvants. But it provides an opportunity for tolerogenic immunotherapies that may harness these mechanisms to more effectively induce tolerance against autoantigens in the setting of autoimmunity. Most current treatments for autoimmunity act through global immunosuppression, rendering patients susceptible to opportunistic infections and deleterious side effects (16). There remains a significant need for antigen-specific immunotherapy (ASIT) strategies that induce selective tolerance against specific autoantigens and autoreactive cells while leaving the remainder of the immune response intact. Mucosal administration of ASIT therefore offers an attractive approach by targeting immune cells in barrier tissues and MALT that are already equipped with regulatory mechanisms to maintain homeostasis (17).

Although well-motivated by the biology of mucosal immunity, delivery of both mucosal vaccines and immunotherapies across mucosal barriers has been a major challenge for development – indeed, a main reason why only a handful of mucosal vaccines and no mucosal ASITs are licensed for clinical use (2, 10, 18). Of the small number of mucosal vaccines that have been licensed, all of them (except the inactivated oral cholera vaccine) are based on live attenuated pathogens that naturally infect mucosal surfaces (2, 19, 20). Yet live attenuated vaccines often face stability and safety concerns, which often prevent their use in immunocompromised individuals. Subunit vaccines, while safer and more stable than traditional live attenuated pathogenic vaccines, tend to exhibit poor immunogenicity and short-lived responses when administered mucosally due to poor uptake. Similarly, despite a long history documenting the efficacy of ‘oral tolerance’ to treat animal models of autoimmunity, this approach has yet to successfully translate to humans because a prohibitively high dose of antigen is required compared to other routes of administration to overcome degradation and limited uptake across mucosal barriers in the gut (21, 22). In both cases, the dose is lost due to poor diffusion across epithelial tight junctions combined with rapid mucociliary clearance and acidic or enzymatic degradation in mucus layers (2). Development of engineering strategies that can overcome barriers to mucosal delivery are thus critical for translation of mucosal subunit vaccines and immunotherapies to the clinic.

Here, we present the current landscape of licensed mucosal vaccines with a focus on emerging engineering strategies that are being developed to enhance mucosal uptake of subunit vaccines and immunotherapies in rodent and primate models. We characterize respiratory mucosal anatomy, NALT and BALT, and their roles in adaptive immunity. The role of IgA and IgA-secreting plasma cells in protection against infection as well as their complex role in autoimmunity are highlighted. In all, by presenting a rationale for targeting NALT/BALT alongside engineering strategies that aim to overcome mucosal barriers to uptake, we hope to share an informed view that could help guide the development of next-generation mucosal vaccines and immunotherapies.

## MALT, germinal centers, and IgA

Analogous to lymph nodes and other secondary lymphoid organs, MALTs serve as a main site for orchestration of antigen-specific immune responses, exhibiting germinal center (GC) formation upon antigenic priming (5). GCs serve two main functions that are critical to a robust immune response following immune activation from infection, vaccination, or autoimmune damage: 1) antibody affinity maturation and 2) B cell clonal expansion (23). B cells exiting the GC are fated to differentiate into long lived plasma cells (LLPCs) or memory B cells (MBCs) (24). Class switching, which was traditionally believed to be a hallmark of the GC, is now thought to occur prior to GC formation but is still a critical process that occurs in the MALT in response to mucosal antigen priming (25). Like those found in peripheral LNs, MALT GCs provide a training ground for a robust high-affinity antibody response through activation of GC B cells and T follicular helper (Tfh) cells that have undergone multiple rounds of antigen presentation, somatic hypermutation (SHM), and affinity maturation leading to differentiation of mature high affinity B cells into memory B cells or plasma cells. Unlike those found in peripheral LNs, however, GC B cells in MALT preferentially class switch toward an IgA isotype, leading to the expansion of both IgG- and IgA-secreting plasma cells to generate a distinct mucosal humoral phenotype (26).

IgA is the most abundant antibody in the mucosa and second most abundant antibody in the serum. Class-switched B cells generate IgA via the T cell-dependent (TD) pathway that is dependent on CD40 engagement and cytokine transforming growth factor-β1 (TGFβ1), or via the T cell-independent (TDI) pathway. The TD pathway occurs in GCs of secondary lymphoid organs and tertiary lymphoid structures, accompanied by affinity maturation and resulting in IgA specialized with high affinities to neutralize toxins and pathogens. The TDI pathway, however, occurs in the lamina propria and can be enhanced by mucosal dendritic cells (DCs) and epithelial cells, resulting in low-affinity IgA specialized to moderate commensals and microbiota in the mucosa (27). IgA is divided into two subclasses: IgA1 is a glycosylated and flexible version of IgA that is more prevalent in the serum and equally distributed across mucosal tissues, while IgA2 is more prevalent in the colon (28). IgA can also be found in three different forms: monomeric IgA, dimeric IgA (dIgA), and secretory IgA (sIgA). Monomeric IgA is secreted by plasmablasts from the bone marrow or lymphoid tissues and exists predominantly in the serum (28). dIgA is secreted by local plasmablasts in the mucosal tissue and becomes sIgA when it is transported across the epithelial barrier to the luminal side by the polymeric Ig receptor (pIgR), where pIgR is cleaved and imparts dIgA with the secretory component (SC) to protect it from mucosal degradation (29). The SC allows sIgA to perform its key protective functions at mucosal sites such as immune exclusion and direct pathogen neutralization (30). Due to its location, unique physiology, and role in IgA production, the NALT and BALT present excellent targets for mucosal vaccines.

## Upper respiratory tract and nasal associated lymphoid tissue

### Anatomy of the nose

**Figure 1:**
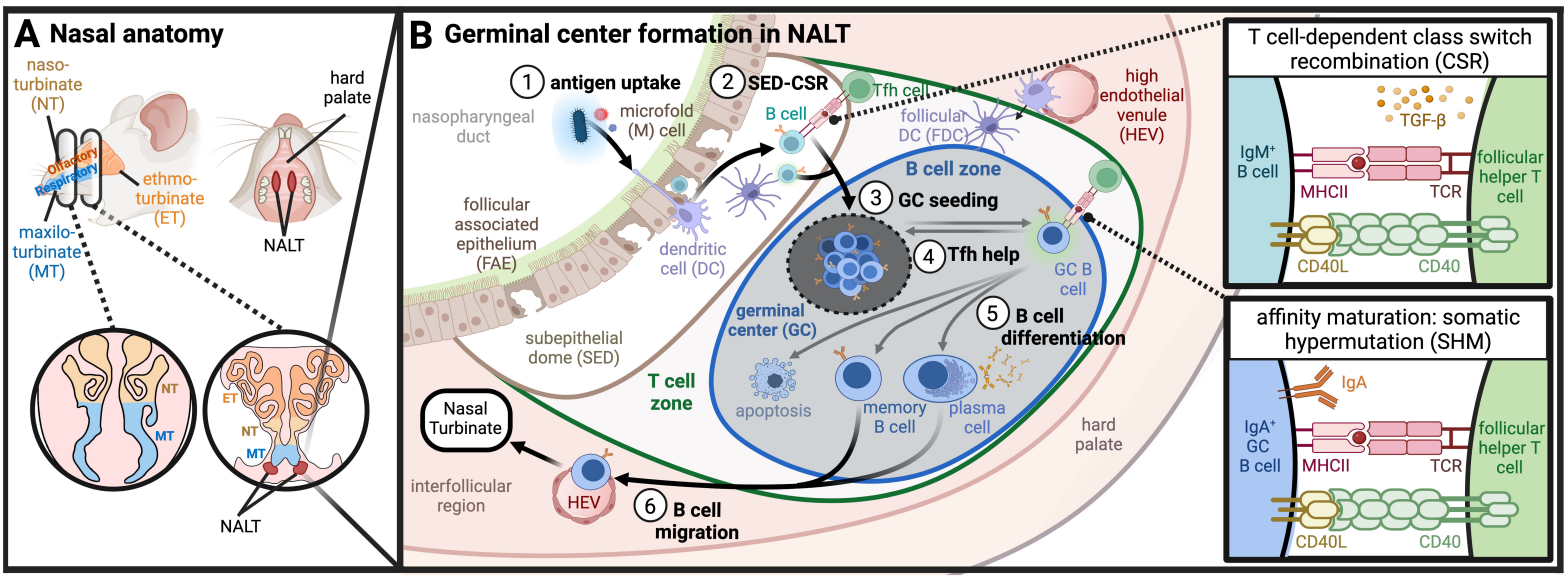
UPPER RESPIRATORY TRACT. **(A)** Nasal anatomy and location of NALT: Murine nasal anatomy consists of the naso-, ethmo-, and maxilo- turbinates (NT, ET, MT, respectively). The nasal-associated lymphoid tissue (NALT) is found medially in the hard palate, positioned proximal to the ethmoturbinates. **(B)** Germinal center formation and immune priming in NALT (1). Antigen uptake across follicle-associated epithelium (FAE) results in (2) activated B cell class switch recombination (CSR) in the subepithelial dome (SED) signaled by DCs or by T cells. Class switched B cells preferentially form IgA through TGF-b signaling, (and (3) seed germinal centers (GCs) in the B cell zone of the NALT where somatic hypermutation (SHM) occurs (4). B cells within the GC compete for T follicular helper (Tfh) cell signaling which results either in the return to GCs or (5) B cell differentiation towards memory B cells, plasma cells, or apoptosis (6). Differentiated B cells migrate through high endothelial venules (HEVs) and lymphatics to turbinates where they reside as IgA-secreting cells. (Created with BioRender.com).

The mammalian nose includes a nasal sinus, paranasal sinus, and nasal associated lymphoid tissue (NALT), with the airway divided along a cartilaginous and bony septum (26, 31, 32). Turbinates are bony, well vascularized projections into the nasal airway lined by mucosal epithelial tissue (33, 34). In humans, the nasal structures are broadly identified as inferior and superior turbinates and maxilloturbinates (31, 35, 36). In mice, the nasal structures are broadly identified as the maxilloturbinate, nasoturbinates, vomeronasal organ, and ethmoturbinates (**Figure 1A**), found in a much more intricate architecture. The vomeronasal organ in mice is encapsulated within the anterior base of the septum and reaches into both nasal compartments, important for the detection of pheromones (37, 38). The naso- and ethmoturbinates compare to the superior turbinates in humans; with the vomeronasal organ, they are considered to function mainly for olfaction (35, 38, 39). Human nasal structures are relatively simple compared to those of rodents, which as obligate nose breathers evolved to rely heavily on the olfactory senses. Human noses instead became more specialized in respiratory function and thus contain significantly less olfactory epithelium. The difference in nasal-olfactory-associated surface area between humans and rodents is stark, on average around 3% in humans compared to 50% in rodents (34). Perhaps more significantly, the complex turbinate surface area in mice has been reported as 150-200 cm^2^ in primates compared to ~2.9 cm^2^ in rodents, which equates to an approximately five-fold higher surface area-to-volume ratio in rodents compared to primates (33). Herein lie two potential functional implications of these anatomical differences: a greater SA:V ratio provides greater opportunity for drug uptake in mice, while greater respiratory epithelial cell composition may provide the main avenue for drug uptake in primates. These marked differences between rodent and primate nasal anatomy may contribute to differences in efficacy observed with intranasal approached when making the jump from small to large animal models, and call for consideration of nasal vaccine uptake in both rodent and primate models during preclinical development. While there is a large body of literature covering traditional pathogenic and vector-based intranasal vaccines in primates and humans (40) (41–43), comparatively little data exists on *intranasal subunit vaccine uptake* in primates and humans. Thus, for the purposes of evaluating uptake, our discussion of anatomy will center around that of laboratory rodents, primarily mice.

### Anatomy of the NALT

In rodents, antigens trafficked across the nasal turbinate epithelium are transported to the NALT, a set of organized mucosal-associated secondary lymphoid tissues that sit along the nasal floor (**Figure 1A**). The NALT can be seen macroscopically as bilateral elongated lymphatic tissue connected dorsal to the hard palate (44). The proximal portions of NALT are found medially in the upper palate, slightly caudal to the ethmoid turbinates (45). When observed from cranial sections of the nose, NALT is commonly described as two “bell-shaped” clusters of densely packed lymphoid cells (39, 45, 46). In humans and primates, the analogous inductive site associated with nasal and oral mucosae is known collectively as the Waldeyer’s Ring, a complex of lymphoid tissues consisting of the nasopharyngeal tonsil, lingual tonsil, palatine tonsils, and the tubal tonsils (36). The Waldeyer’s Ring has been reviewed in depth elsewhere (47). Importantly, the NALT and Waldeyer’s Ring both serve as mucosal inductive sites where antigen-specific immune responses against inhaled pathogens are orchestrated (2, 33, 48). This includes activation of antigen-specific T cells and B cells, licensing of lymphocytes and plasma cells for mucosal homing, and priming of humoral immunity (both systemic IgG and mucosal IgA responses) through formation of GCs. Indeed, NALT is a main source of nasal-resident IgA-secreting plasma cells (49).

More specifically, the NALT consists of a subepithelial dome (SED) and underlying follicular regions where GC formation takes place (**Figure 1B**). Atop the SED sits a specialized cluster of epithelial cells called the follicular associated epithelium (FAE). The FAE is a morphologically distinct section of epithelium that displays a periodic lack of ciliated cells due to an increased frequency of DCs and microfold cells (M cells) that sample the lumen for antigen (39, 45, 50, 51). M cells are terminally differentiated epithelial cells that are specialized to funnel antigen from the lumen; they sparsely line the nasal cavity but are found in higher density in the turbinate epithelium and FAE (52–55). Just below the FAE sits the SED of organized mucosal lymphoid tissue where antigen transported by M cells and sampling DCs is handed off to other antigen presenting cells (APCs) such as DCs, macrophages, and B cells, before transitioning into a follicular region segregated into T cell and B cell zones (39).

The SED is thought to serve as an active site of B cell class switch recombination (CSR), thought to occur prior to GC formation in either a TD or TDI mechanism (25). B cells expressing activation-induced cytidine deaminase (AID), an enzyme critical for CSR and SHM, are observed to increase substantially in the SED followed by dissemination of GC seeding B cells (49). B cell priming in the NALT SED mirrors the mechanisms observed in Peyer’s patches (PPs) (56–58). In contrast to PPs in the gut and LNs in the periphery, however, the NALT is already segregated into a B cell zone and minimal outer regions of T cell zones at steady state. Upon infection, CD4^+^ T cells undergo rapid expansion, essential for robust IgA response through TD CSR (49, 50). High endothelial venules (HEVs) in interfollicular regions provide pathways for antigen transport and cell migration into or out of the follicles, critical for NALT development and priming adaptive immune responses (59, 60). While the importance of afferent versus efferent lymphatics in the NALT remains unclear, antigen drainage to cervical LNs is dependent on NALT lymphatics (51, 61, 62), and cervical LNs are also essential for NALT organ development (63).

### Organogenesis

Despite structural and functional similarities, mouse NALT develops independently of the transcriptional and signaling regulators observed in lymph nodes or PPs. In PPs, lymphoid tissue inducer cells are observed prenatally resulting in mice born with morphologically mature PPs (64, 65). In contrast, NALT develops entirely postnatally; not until 7-10 days post-birth do lymphoid tissue inducer cells initiate development of the NALT, which is not fully developed in mice until 8 weeks of age (66, 67). This timeline should be factored into preclinical mouse intranasal immunization studies, which, if looking to target NALT, should be carried out with mice at least 8 weeks old. While NALT organogenesis is still not fully understood, its development is thought to be dependent on the postnatal presence of commensal bacteria and antigen stimulation through pattern recognition receptors (PRRs) (59, 67–70). So far, Id2 is the sole transcriptional regulator shown to control initiation of NALT organogenesis (71), although developmentally critical cell recruitment through HEVs depends on CXCR5 signaling (59). In general, it is postulated that postnatal tissue organogenesis creates a form of “flexibility” that significantly factors into the developmental adaptability of NALT (72).

## Lower respiratory tract and bronchus associated lymphoid tissue

### Anatomy of the lungs and BALT

**Figure 2:**
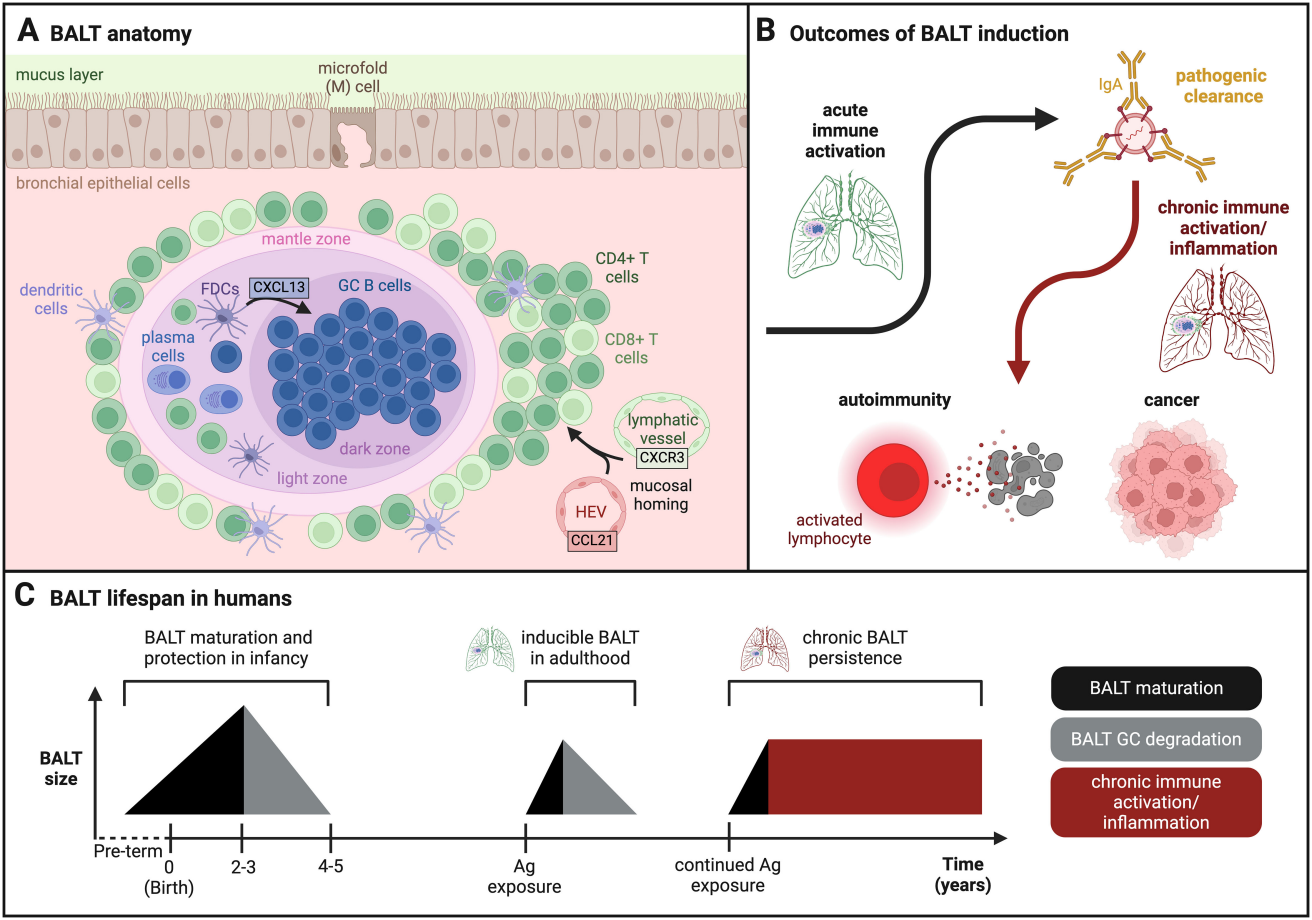
LOWER RESPIRATORY TRACT. **(A)** BALT follicular anatomy in lungs: Lymphocytes home to the bronchus subepithelial space via lymphatic vessels and HEVs where they form an ectopic lymphoid structure known as the bronchus associated lymphoid tissue (BALT). This tissue is characterized by densely packed B cell follicles with distinct germinal center (GC) behavior. **(B)** Outcomes of BALT induction: The BALT plays a positive protective role against respiratory pathogens via antibody (IgA, IgG) clearance. However, during prolonged chronic inflammation, BALT is associated with harmful conditions such as autoimmunity and cancer. **(C)** Timeline of BALT formation and degradation in humans: Infant BALT often forms in fetal lungs, reaches maximum size by year two to three, and fully degrades by year four or five. As an adult, BALT can be transiently induced by respiratory challenge but will degrade following clearance and resolution. However, chronically induced BALT that persists over time is a marker for harmful chronic immune activation and inflammation in the bronchials. (Created with BioRender.com).

The lower respiratory tract presents another attractive mucosal site for immunomodulation through antigen uptake into underlying BALT (**Figure 2**). BALT is continuously present in some mammals like rats and rabbits, but only transiently present as ‘inducible BALT’ in mice and humans (73, 74). For the purposes of this review, we will typically refer to both as ‘BALT’. Initially discovered in the late 1800s, the lymphatic functionality of BALT remained unclear for over 100 years until new insights were provided late in the 20^th^ century (75, 76). BALT was originally thought to be analogous to gut associated lymphoid tissue (GALT) in the intestines, as both tissues form in the mucosal epithelium and serve as sites for APC priming and expansion of mature B/T lymphocytes (76). For example, B cells in BALT express high levels of IgA due to abundant antigen exposure in the lungs (77), and T cells traffic to BALT from the spleen in response to respiratory infections such as influenza (78). However, unlike classical mucosal lymphoid tissues like Peyer’s Patches in the intestines or NALT in the upper respiratory tract, BALT is not constitutive throughout life nor does it always present with the same organization.

BALT is characterized as a tertiary lymphoid tissue that forms in the bronchial epithelium adjacent to the airway and pulmonary arteries. divided into distinct B cell and T cell zones supported by a stromal cell network (**Figure 2A**). Unlike the NALT and GALT, SED is not always present underlying the airway surface in BALT. Rather, to be considered a mature BALT, tertiary or ectopic lymphoid tissue must exhibit defined B cell follicles colocalized with follicular dendritic cells (FDCs) (79, 80). BALTS form when lymphocytes traffic from the blood or other secondary lymphoid tissues (lymph nodes, spleen) via lymphatic vessels and HEVs to the site of antigen exposure during mucosal homing (81). Immune cells are recruited from the pulmonary tract via CCL21 chemokine signaling (79, 82), while lymphocytes home from the spleen and lymph nodes via CXCR3 signaling (83, 84). BALT follicular organization is driven by FDCs and lung fibroblasts that secrete CXCL13, an essential chemokine that directs the organization of B cells into densely packed GCs within the follicle (**Figure 2A**) (85). The B cell follicle is surrounded by T cells with a Th2 phenotype. Tfh cells provide B cell help to stimulate GC B cell clonal expansion and antibody affinity maturation. In humans, this response is only induced in mature BALTs in response to specific respiratory antigens, for example, triggering IgG/IgA responses against influenza (82, 85, 86) or IgE responses against allergens (**Figure 2B**) (85, 87, 88). Thus, the BALT GC response is highly adaptive and specific when faced with respiratory antigens.

### Inducible BALT

The term ‘inducible BALT’ (iBALT) was coined by Moyron-Quiroz et al. in 2004 to describe the ability of mice that lacked traditional lymphoid organs such as spleen, lymph nodes, and PPs to generate a lymphocyte response through formation of iBALT after exposure to influenza antigen (80). In these mice, iBALT acted as a replacement for the missing lymphoid organs, mounting an immune response against respiratory pathogens from within the lungs that resulted in lower mortality and weight loss than mice lacking iBALT. Immune activation of lymphocytes in iBALT was significantly lower (~20%) than that of wild-type mice after 10 days; yet, this response was considered sufficient as it was localized to the lungs and avoided systemic inflammation that often leads to weight loss and death in normal mice. Moyron-Quiroz et al. argued that upon induction of iBALT, lung-targeted vaccines against respiratory pathogens may be an effective strategy for preventing off-target immune damage from chronic inflammation. Additional discussion of iBALT as a training ground for T and B cell adaptive immune responses and its role in protection versus pathogenesis of pulmonary disease is reviewed elsewhere (89, 90).

### Persistent BALT

In species such as humans and mice, the presence of BALT in adults can be associated with beneficial immune clearance but can also indicate chronic inflammation (**Figure 2B**) (80). Ectopic lymphoid follicles are often found in patients with autoimmune conditions such as rheumatoid arthritis (RA) and Sjogren’s syndrome (SS). Tissues from these patients exhibit several BALT-like features, such as GC B cells specific for biotinylated human IgG and RA autoantigens. RA patients with developed BALT exhibit elevated levels of autoantibodies such as those against cyclic citrullinated peptide (aCCP Ab) in bronchoalveolar lavage fluid, and more severe lung fibrosis due to α-SMA-expressing myofibroblast density near BALT GCs (85). BALT induction also occurs at a higher frequency in farmers who develop hypersensitivity pneumonitis from repeated exposure to allergens from barn and animal dander. When sampled, BALT was observed at various stages of development in these patients, although there was no causal relationship found between the severity of the disease and level of BALT organization (87).

Along with higher incidence in autoimmune disease, chronic BALT formation carries some oncological risk (**Figure 2B**). For example, prolonged inflammation serves as the catalyst for development of non-Hodgkin’s lymphoma in MALT, often stemming from bacterial infection or chronic autoimmune conditions (91). Most MALT lymphomas are preceded by bacterial infection by organisms such as *Helicobacter Pylori* (HP) or autoimmune diseases such as SS (92). It seems the transient and retractable nature of inducible BALTs in adults is key for effective immune protection and homeostasis in the lung. Acute immune activation allows for pathogenic clearance by selectively triggering inflammation in tissues where and when it is needed, while chronic activation and persistent BALT can lead to harmful inflammation with increased incidence of autoimmunity and cancer (**Figure 2C**). As such, chronically inflamed BALTs may present a target for immunotherapies to combat autoimmune conditions and lymphomas by reducing inflammation.

### BALT in early life immunity

Organized BALT follicles containing GC B cells and FDCs are observed in the lung lymphoid tissue of children starting at a young age. In infants and young children with naive immune systems, ‘early-life BALT’ provides protection from respiratory infections, enabling quick recovery without lifelong consequences (93) while playing an active role in establishing lung immune memory early in life (94). Early-life BALT was first discovered by Gould et al. in 1993 while examining histologically stained lung sections of fetuses and infants who succumbed to disease. They discovered lymphoid tissue in areas of developing lymph nodes and, in particular, immune cell aggregates in branch points of the bronchial epithelium that indicated functional BALT (95). For many of these infants, formation of BALT correlated with incidence of infection and disease: 4 of 4 (100%) infants with ascending infection and 11 of 15 (73%) infants with sudden infant death syndrome (SIDS) exhibited BALT, while only 4 of 20 (20%) infants with no evidence of infection exhibited BALT. Matsumoto et al. built upon this finding when they observed that BALT develops during the first year of life, achieves maximum size around two to three years of age, and degrades between years four and five before disappearing altogether (**Figure 2C**) (93). At this time, GCs in the BALT can no longer be sustained and lymph nodes take over as the primary site of GC priming for the lower respiratory tract. During years of peak tissue size, BALT GCs express a high level of activity and B cell specificity. A significant portion of these BALT GC B cells become mature IgA^+^ class switched memory B cells that express CD95 (activation marker) and CD69 (tissue residency marker) (81). Class-switched B cells protect against a variety of respiratory threats, producing IgG against seasonal coronavirus, herpesviruses, and polio antigens, and IgA against respiratory syncytial virus (RSV), seasonal coronavirus, and metapneumovirus. The effectiveness of pathogenic clearance and memory establishment in early life BALT suggests this tissue may also serve as an attractive site for targeted respiratory childhood vaccines to encourage more comprehensive, long-lasting immunity.

## Mucosal barriers and uptake in the respiratory tract

**Figure 3:**
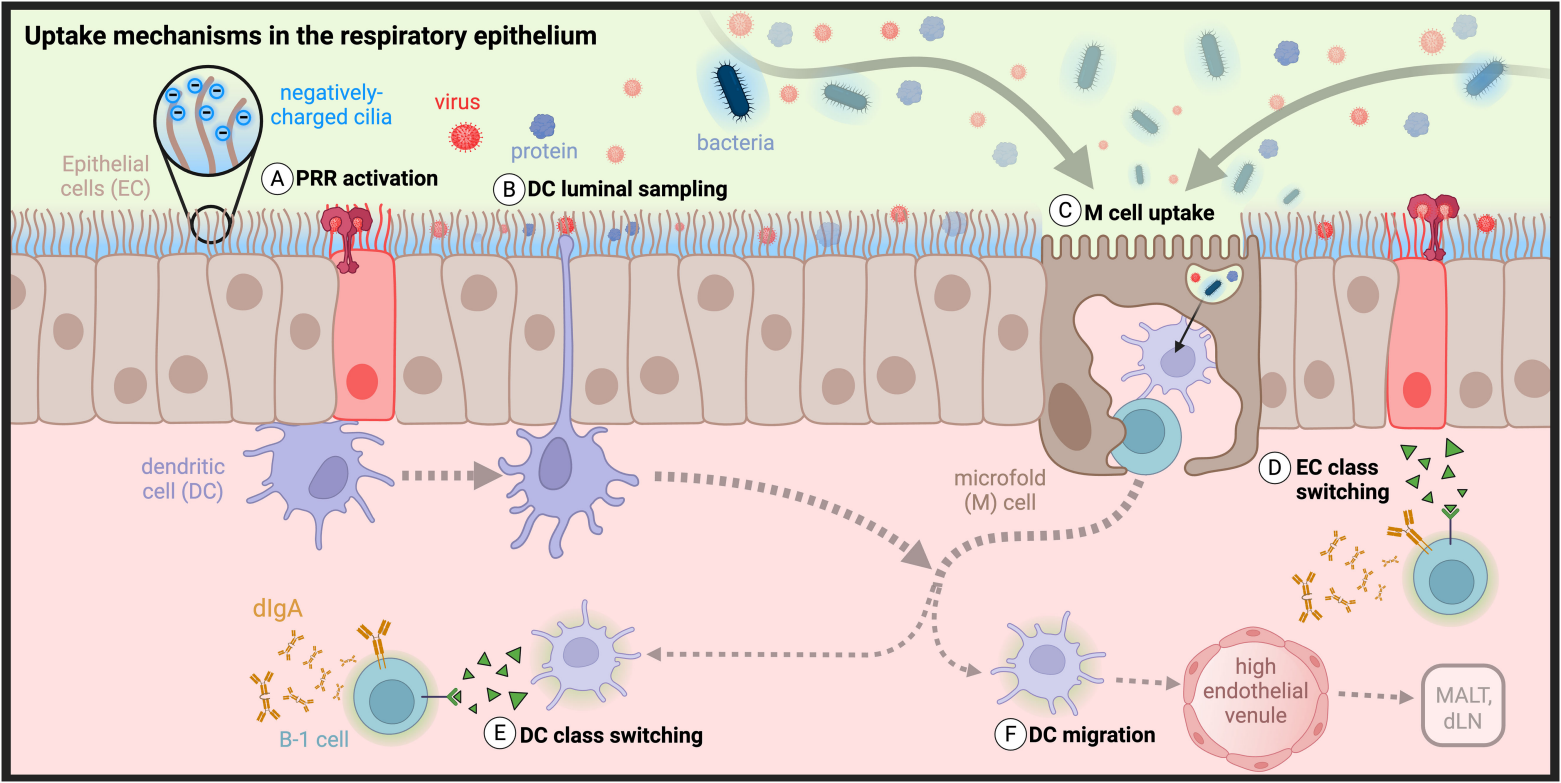
Barriers and uptake mechanisms in the respiratory epithelium: The transport of antigens across respiratory epithelial cells (ECs) is controlled through dense, negatively charged cilia that repel negatively charged antigens to prevent adhesion. **(A)** ECs express pattern recognition receptors that signal to dendritic cells (DCs) the presence of infection and **(B)** triggers them to extend transepithelial dendrites (TEDs) to sample the lumenal space. **(C)** Antigen uptake also occurs by microfold (M) cells. Class switch recombination in B cells can be induced by **(D)** ECs or **(E)** DCs, followed by **(F)** potential DC migration to MALT or draining cervical or mediastinal lymph nodes (dLNs). (Created with BioRender.com)| .

A major obstacle to the development of mucosal vaccines and immunotherapies is achieving sufficient delivery of antigen and other drug components across mucosal epithelial barriers to the underlying MALT (**Figure 3**). Thus, elucidating mechanisms of antigen uptake in addition to activation in the respiratory mucosa may advance the development of more effective mucosal vaccines and immunotherapies. Here we review mucosal barriers in the upper and lower respiratory mucosae and three main cellular components that play a role in active uptake across them: epithelial cells, M cells, and DCs.

### Uptake by epithelial cells

Mucosal surfaces are lined with epithelial monolayers formed by intercellular tight junctions that largely prevent macromolecular passive uptake by diffusion (**Figure 3**) (96). As the primary targets of most invading pathogens, epithelial cells have evolved numerous mechanisms to exclude bacteria, viruses, and other organic and inorganic particles from crossing their boundary. First and foremost, nasal epithelial cells are held together by strong cell-cell interactions called apical junctional complexes, formed by tight junctions and adherens junctions between neighboring membrane proteins. Tight junctions are not merely passive structural elements, instead acting as dynamic and reversible participants of exclusion and selective uptake (97–99). Viruses have evolved strategies to target tight junctions as sites of epithelial invasion, necessitating more active methods of primary protection by innate and adaptive immune cells, recently reviewed by Linfield et al. (100–105). Some of these strategies have been co-opted by researchers to overcome challenges of intranasal delivery (98–100, 106–108).

Respiratory epithelial cells are further protected by a layer of 7-10µm thick viscoelastic mucus secreted by specialized epithelial goblet cells, responsible for efficient mucociliary clearance (109). Secreted mucus is a complex, immunoactive, and proteolytic mixture that is patrolled by innate cells and contains IgA as well as degradative enzymes (110, 111). In humans, the nasopharynx alone secretes an estimated 20-40 mL of mucus per day, accounting for the removal of 80-100% of particles between 4-12.5µm in size. Epithelial cells are densely covered by highly functionalized cilia, immune-exclusionary appendages that beat asynchronously about 1000 times per minute, in order to cycle mucus across the epithelial surface and wash away excluded pathogenic material (112). The importance of mucosal cycling is highlighted by symptoms associated with a loss-of-function: mice deficient in tubulin polyglutamylation (*Ttllk1-*KO) exhibit an accumulation of mucus throughout the nasal cavity and exhibit dysfunction of nasal transport mechanisms (113). Mucus transport is further aided by functionalization of cilia and microvilli with mucin glycoproteins that can be found in membrane-bound (MUC1, MUC4, and MUC20) or polymeric form (MUC5AC and MUC5B) (109). Oligosaccharides line the peptide backbone of mucins resulting in a “bottle-brush” structure with a densely packed negative charge (**Figure 3**) (114, 115). Mucins’ negative charge results in their observed lubricity and helps to maintain mucus adherence for effective ciliary-mediated movement (116). Additionally, their high frequency results in the buildup of a net negative charge, creating an electrostatic barrier that can repel or trap charged bacterial and viral species such as *E. Coli* and adenovirus (109, 114, 117–119).

Beyond physical exclusion of potential invaders, epithelial cells are active participants of immunity through innate signaling, trafficking of sIgA via pIgR, and bidirectional transcytosis of IgG and albumin via the neonatal Fc receptor (FcRn) (48, 120–124). Innate activation of epithelial cells is triggered by pathogen- and damage-associated molecular pattern (PAMP/DAMP) recognition through PRRs such as toll-like receptors (TLRs), initiating multiple immune pathways that include IgA class switching (**Figure 3A**) (119, 125–128). Epithelial TLR activation results in variable activation of NF-κB and IFN signaling pathways followed by secretion of inflammatory cytokines such as TSLP, GM-CSF, IL-33, IL-25, IL-6, IL-8, and IFN-B (97, 129, 130). This results in the activation of a frontline immune response through recruitment of innate immune cells such as macrophages, neutrophils, and DCs (50, 125, 126).

### Uptake by dendritic cells

Given the barriers to diffusive uptake, transport of molecules from the nasal and pulmonary lumen across the respiratory mucosal epithelium is thought to be restricted mostly to active transport by differentiated M cells and lumenal sampling DCs (131, 132). DCs are known as professional antigen presenting cells (pAPCs) due to their ability to activate both naive CD8^+^ and CD4^+^ T cells through MHCI and MHCII presentation, respectively. However, their diverse functions also include antigen recognition, uptake, and trafficking; innate immune signaling; and immune activation and suppression (50, 133). DCs can be found in both lymphoid and nonlymphoid tissue where they play a central role in activating or suppressing the immune response, and also in mucosal epithelium where they play a central role in sampling antigen from the lumen (**Figure 3B**). Each case highlights their importance as a target for vaccines and immunotherapies.

DC function depends on tissue, inflammatory context, and subtype, which are broadly classified into plasmacytoid DCs (pDCs) or conventional DCs (cDCs). Through either recognition of TLRs or phagocytosis of dead/dying cells, pDCs recognize intracellular viral DNA or RNA and secrete inflammatory cytokines to prime an immunogenic response, while cDCs recognize pathogens and cross-present antigens to CD4^+^ and CD8^+^ T cells (86, 126, 133–139). pDCs have also been implicated in the suppression of inflammation in the lungs and the generation of inducible Tregs (iTregs) (140). A comprehensive overview of DC phenotypes and function in the nose is described in depth by Lee et al. (50). In brief, conventional and inflammatory DCs are found throughout the nasal epithelium, while conventional and plasmacytoid subsets are found in the NALT. Immune-activating DCs tend to migrate from the nasal epithelium to the NALT and to cervical LNs leading to the expansion of CD4+ T cells. Subepithelial-residing DCs rarely sample the lumen spontaneously, instead requiring an epithelial cell “alert” to trigger extension of transepithelial dendrites. Epithelial cells utilize CCL20-mediated DC recruitment and require DC expression to regulate the frequency of these transepithelial extensions. DCs can be further activated directly or indirectly through epithelial cell-DC crosstalk pathways involving growth factors (GM-CSF, GCSF, VEGF), TSLP, and BAFF, as well as chemokine-mediated DC recruitment (126, 141–144). DCs also play a critical role in antigen sampling and trafficking in the lungs, where live cell imaging shows that DCs can acquire antigen in the pulmonary airway and then traffic through afferent lymphatics to present antigen to T cells in the BALT (145).

### Uptake by microfold cells

M cells are found scattered throughout the lining of the FAE of NALT and Peyer’s Patches where they sample antigen from the mucosal lumen and hand off to underlying APCs in the SED (**Figure 3C**). M cells are also found in regions of nasal and intestinal epithelium that are distant from MALT, such as the nasal turbinates and intestinal villous epithelium, respectively. Given their relative abundance in the FAE of the NALT, M cells are considered a primary avenue for transepithelial antigen delivery to the NALT, known as professional transporters due to their dominant role as mediators of luminal antigen transcytosis (52, 146, 147). Suited to their role in antigen uptake, M cell apical membranes exhibit wavy indentations known as microfolds instead of cilia. This lack of cilia is accompanied by a loss of mucins and therefore a relatively neutral apical surface charge (118). When surrounded by negatively charged cilia on neighboring epithelial cells, this neutral membrane creates an “electrostatic funnel” that directs negatively charged antigens toward M cell mediated uptake. Indeed, a direct relationship has been observed between M cell uptake and nanoparticle electrostatic charge in combination with buffer ionic strength. Potential receptor-mediated uptake may therefore be superseded by the role of charge in targeting M cells (148, 149). Finally, the basolateral side of M cells creates a “pocket” that primarily houses APCs and lymphoid cells, where signaling takes place between M cells, APCs, and lymphocytes that can induce polyclonal IgA responses (52, 146). For this reason, the M cell pocket is considered an extension of the GC in PPs, however a parallel mechanism has not yet been confirmed in nasal or pulmonary epithelium (150).

The role of M cells in mediating antigen uptake at mucosal surfaces in the NALT and PPs is reviewed in more depth elsewhere (149). M cells have also been observed in the lungs in FAE overlaying BALT (151). However, as well-defined FAE and SED are not always present in BALT, M cells may be one potential pathway for antigen uptake in the lungs but are likely not a primary pathway. In pulmonary tissues where M cells are not found, antigen uptake may occur instead by mechanisms such as infection or DC trafficking through afferent lymphatics (73).

## Mucosal vaccines in the clinic: currently licensed or in clinical trials

Mucosal surfaces are the first entry point for most pathogens, such as HIV, SARS-CoV-2, influenza, cholera, tuberculosis, and rotavirus. In the year 2019 alone, lower respiratory tract infections were responsible for approximately 2.5 million deaths worldwide (152). For effective protection and management against many of these infections, a coordinated response involving both serum IgG and mucosal IgA in tandem is often required, with immunity in mucosal barrier tissues as a first line of defense (153). In light of the recent COVID-19 pandemic that has seen continued waves of transmission with evolved variants, there is a renewed interest in developing ‘next generation’ mucosal vaccines that elicit immunity in mucosal barrier tissues and are capable of blocking infection and transmission (3, 154–156). For example, a recent preclinical study by Bull et al. showed that intranasal immunization with Bacillus Calmette–Guérin (BCG), the only licensed parenteral vaccine against tuberculosis (TB), offers improved protection by significantly increasing antigen-specific lung Trm cells in mice (157). Another preclinical study comparing intranasal and intramuscular administration of a chimpanzee adenovirus-vectored vaccine encoding a prefusion stabilized spike protein from SARS-CoV-2 showed that animals receiving an intranasal dose exhibited reduced viral load in the respiratory tract (158). These findings highlight that mucosal vaccines targeting the respiratory tract hold potential as an effective immunization strategy, both through the establishment of frontline humoral immune protection and also, more recently recognized, establishment of tissue resident memory T cells.

**Figure 4:**
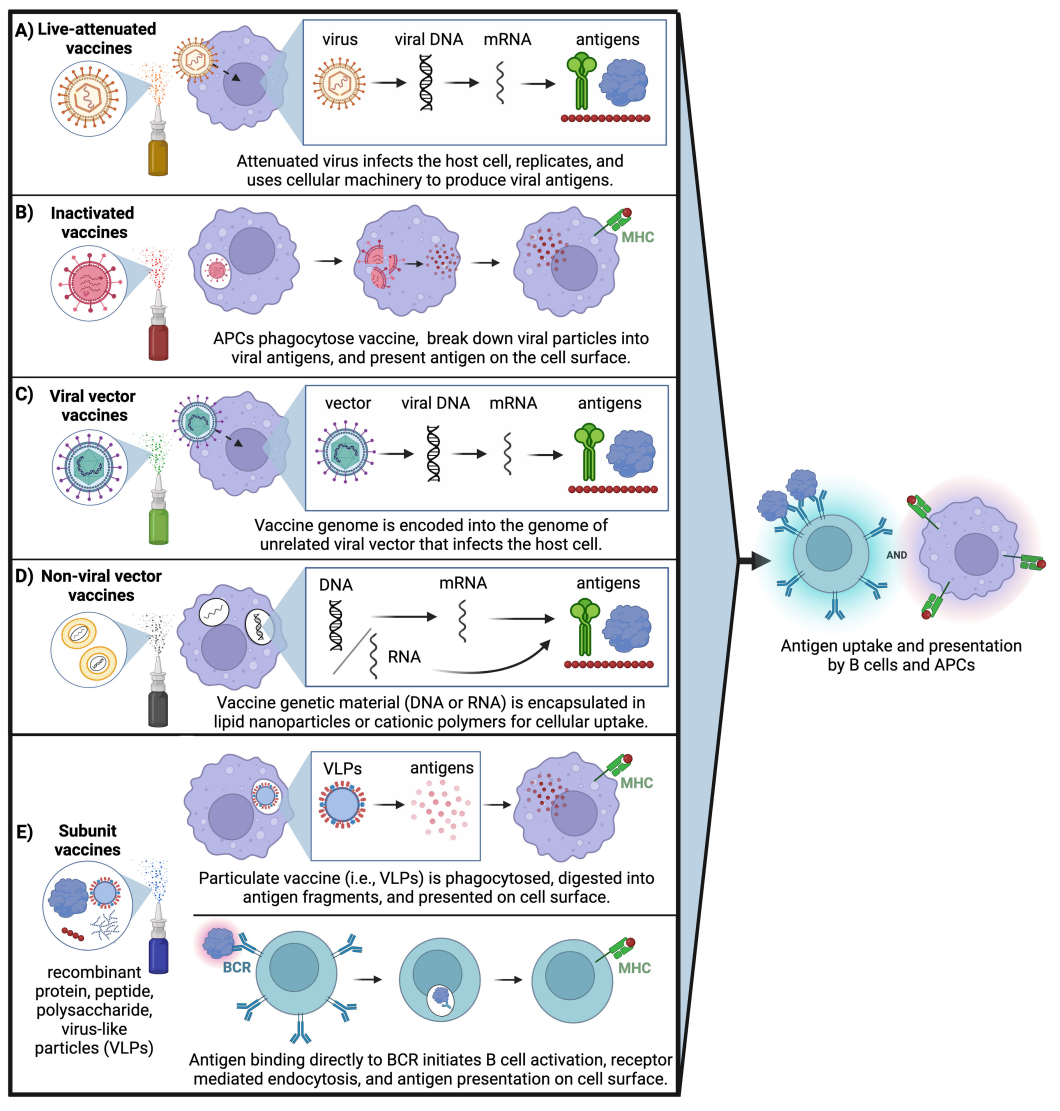
Mucosal vaccine approaches: All mucosal vaccines that are clinically approved are based on live attenuated or inactivated pathogens; however, ongoing clinical trials are investigating other strategies to overcome challenges associated with pathogenic vaccines. **(A)** Live attenuated virus infects the host cell, undergoes replication, and uses cellular machinery to produce viral antigens. **(B)** Inactivated virus is phagocytosed by APCs, broken down into viral antigens, then presented on the cell surface. **(C)** Vaccine genome is encapsulated into an unrelated viral vector that infects the host cell then uses cellular machinery to produce viral antigens. **(D)** Genetic content from the vaccine (either DNA or RNA) is encapsulated in lipid nanoparticles or cationic polymers for cellular uptake followed by translation of viral proteins. **(E)** Subunit vaccines can utilize two delivery mechanisms: Particulate vaccine containing virus-like particles is phagocytosed by APCs and digested into viral antigen fragments followed by antigen presentation on the cell surface. Or, recombinant and/or particulate subunit vaccines can directly bind to the B cell receptor (BCR) on the surface of B cells followed by receptor-mediated endocytosis and antigen presentation on the cell surface. (Created with BioRender.com).

Yet despite the need for vaccines that can promote more comprehensive mucosal immunity, only a handful of mucosal vaccines have reached licensure (**Table 1**). All of these except the inactivated oral cholera vaccine are live-attenuated vaccines, which have historically been used for mucosal immunization because they naturally infect mucosal surfaces and can promote robust immunity. Live-attenuated vaccines contain whole virus in a weakened state that infects host mucosal epithelial cells, replicates, and uses cellular machinery to produce viral proteins (**Figure 4**). Inactivated vaccines contain a fixed form of virus that is taken up by antigen presenting cells, broken down into viral antigens, and then presented on the cell surface for immune activation. Currently, live-attenuated influenza type A/B vaccine (FluMist) is the only licensed intranasal vaccine that specifically targets the respiratory tract. However, production, stability, and safety issues with live-attenuated vaccines are common. They cannot be used to immunize individuals with naturally weakened immune systems such as infants and elderly, or individuals who are immunocompromised such as pregnant women, transplant recipients, and patients receiving immunosuppressive autoimmune or cancer therapies (167). Additionally, inactivated vaccines can undergo epitope alteration during the inactivation process, which raises safety concerns for these populations (168).

**Table 1:** Clinically approved and licensed mucosal vaccines for use in humans.

Type	Pathogen (Disease)	Vaccine	Administration Route	Approval year	References
Live-attenuated	lnfluenza A/B viruses (Influenza)	FluMist™	Intranasal	2003	(159)
	Vibrio cholerae (Cholera)	Vaxchora™	Oral	2015	(160)
	Rotavirus (Diarrheal)	RotaTeq™	Oral	2006	(161)
		Rotarix™	Oral	2008	(161)
	Salmonella typhimurium (Typhoid)	Typhi Vivotif™	Oral	2013	(162)
	Poliovirus (Polio)	Oral polio vaccine (OPV™)	Oral	1961	(163)
Inactivated	Vibrio cholerae (Cholera)	Dukoral™	Oral	2003	(164)
		Shanchol™	Oral	2013	(165)
		Euvichol™	Oral	2013	(165, 166)

The challenges associated with live-attenuated and inactivated pathogenic vaccines have prompted a shift in parenteral vaccines towards recombinant protein-, peptide- or polysaccharide-based subunit vaccines that tend to be safer, more stable, and easier to manufacture (**Figure 4**). While there are no subunit mucosal vaccines currently licensed for clinical use, the Center for Genetic Engineering and Biotechnology in Havana is developing a protein subunit-based intranasal booster dose for SARS-CoV-2 that is currently in Phase II clinical trials (**Table 2**) (183). This vaccine is synthesized using recombinant receptor binding domain (RBD) protein from SARS-CoV-2, the portion of the spike protein that is responsible for binding the ACE-2 receptor to gain entry into host epithelial cells.

**Table 2:** Mucosal vaccines currently in clinical trials for respiratory pathogens.

Type	Pathogen	Vaccine	Route	Antigen	Trial Phase	Clinical trial identifier	References
Live-attenuated or inactivated	SARS-CoV-2	COVI-VAC (Codagenix)	Intranasal	Live attenuated SARS-CoV-2	Phase I	NCT04619628	(169)
		DelNS1-2019-nCoV-RBD OPT1 (University of Hong Kong)	Intranasal	Live attenuated influenza virus (LAIV) encoding SARS-CoV-2 receptor binding domain (RBD)	Phase III	ChiCTR2100051391	(156, 170, 171)
	RSV	RSV ΔNS2 Δ1313 I1314L	Intranasal	Live attenuated RSV	Phase II	NCT03916185	(172)
		MV-012-968 (Meissa Vaccines)	Intranasal	Live attenuated RSV with F, SH, and G virulence gene modifications	Phase I	NCT04444284	(173)
	Influenza	BPL-1357 (National Institute of Allergy and Infectious Diseases)	Intranasal	Beta-propiolactone (BPL)- inactivated whole virus	Phase I	NCT05027932	(174))
Non-replicating viral vector		BBV154 (Bharat Biotech)	Intranasal	Adenoviral vector encoding the spike protein	Phase I	NCT04751682	(175, 176)
		PIV5 (CVXGA1)	Intranasal	Parainfluenza virus 5 (PIV5) expressing the SARS-CoV-2 spike protein	Phase I	NCT04954287	(177, 178)
	SARS- CoV-2	VXA-CoV2-1.1 S (Vaxart)	Oral	Adenoviral (Ad5) vector encoding S and N proteins from SARS-Cov-2	Phase II	NCT05067933	(179)
		hAd5-SFusion+ N-ETSD (ImmunityBio)	Oral + Subcu.	Adenoviral (Ad5) vector encoding the S-fusion domain and N protein	Phase I	NCT04732468	(180)
		AdCOVID (Altimmune)	Intranasal	Adenoviral vector encoding RBD of S1 spike protein	Phase I	NCT04679909	(181)
	Influenza	VXA-A1.1 (Vaxart)	Oral	Adenoviral vector encoding H1N1 hemagglutinin (HA) protein	Phase II	NCT00197301	(182)
Protein subunit	SARS-CoV-2	MAMBISA/CIGB 669 (CIGB, Havana)	Intranasal (booster dose)	Monomeric receptor binding domain (RBD) of S1 spike protein	Phase II	RPCEC00000345	(183)

Other mucosal vaccines currently in clinical trials, summarized in **Table 2**, consist of live-attenuated/inactivated pathogenic vaccines or nucleic acid-based vaccines. Candidate vaccines for SARS-CoV-2 include live-attenuated COVI-VAC™ and replicating viral vector DelNS1-2019-nCoV-RBD OPT1™ developed at the University of Hong Kong, currently in Phase I and III trials, respectively. DelNS1-2019-nCoV-RBD OPT1™ uses a live-attenuated influenza virus (LAIV) that encodes for cell surface expression of SARS-CoV-2 RBD (156, 170, 171). COVI-VAC™ uses SARS-CoV-2 virus attenuated by codon pair deoptimization (169). A similar strategy of codon deoptimization is being used to develop live-attenuated RSV mucosal vaccines by non-structural NS1 and NS2 virulence gene modifications (173). Lastly, an inactivated Influenza vaccine called BPL-1357™ is currently in Phase I trials (174).

Nucleic acid-based vaccines involve the delivery of a viral genome into host cells, which then use cellular machinery to synthesize and express copies of viral protein antigens on the cell surface (**Figure 4**). Many mucosal vaccines for SARS-CoV-2 that are in trials such as the BBV154™, PIV5™, VXA-CoV2-1.1-S™, and hAd5-SFusion+ N-ETSD™ utilize this mechanism of non-replicating viral vector. In most cases, mucosal SARS-CoV-2 vaccines are being evaluated as an intranasal booster to follow the intramuscular mRNA COVID vaccine series. For example, in preclinical studies, PIV5 elicited broadly neutralizing antibodies and functional protection against multiple SARS-CoV-2 variants of concern as a single intranasal dose; when administered as a booster dose following two doses of intramuscular mRNA COVID-19 vaccine, it promoted higher levels of cross-reactive neutralizing antibodies and greater protection against viral challenge than three mRNA doses (177). Yet while they tend to be safer and better tolerated than live-attenuated or inactivated vaccines, nucleic acid-based vaccines can still face challenges with breadth and longevity of immune response (184), and contain a risk for insertional mutagenesis that can damage DNA over time and increase the likelihood of mutations in tumor suppressor genes (185).

## Preclinical mucosal vaccine approaches in development

**Figure 5:**
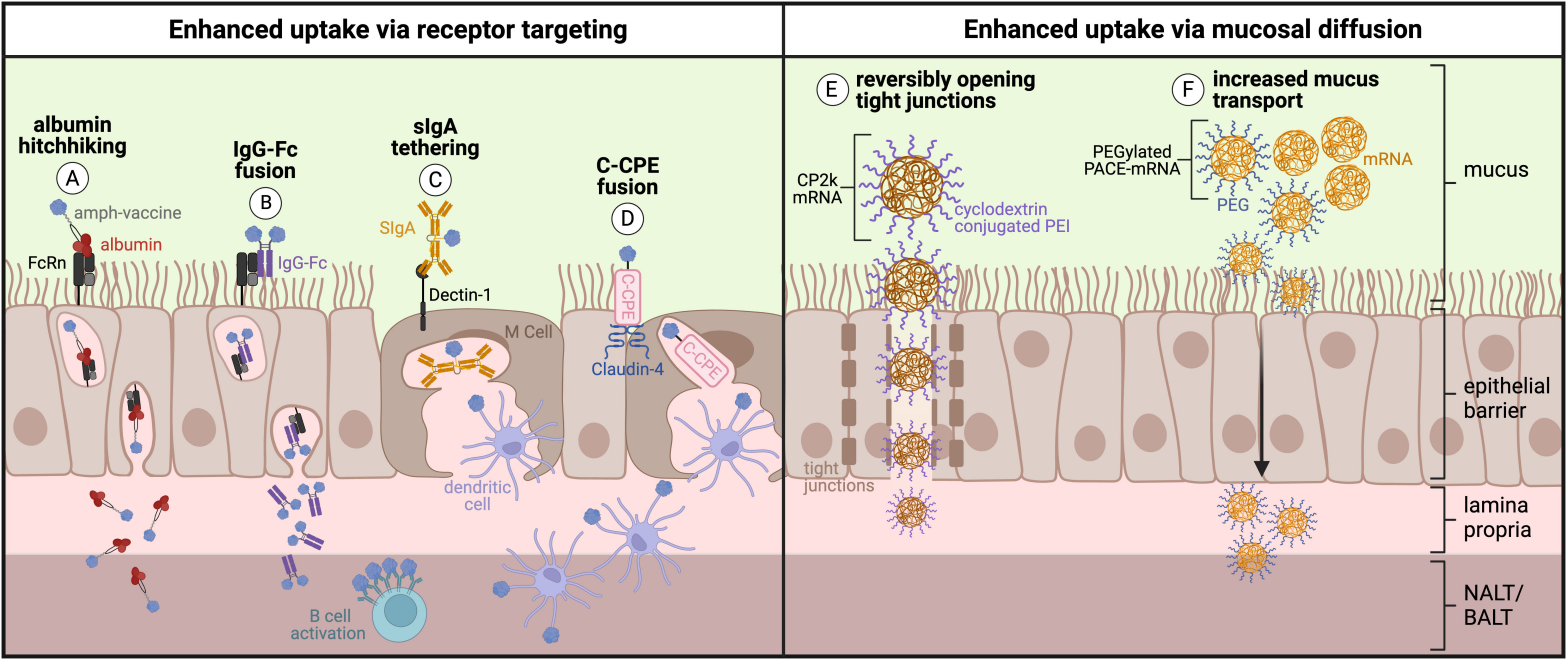
Engineering approaches to enhance active or passive uptake across the respiratory mucosa: **(A)** Protein/peptide antigens conjugated to an amphiphile tail that ‘hitchhike’ on albumin and **(B)** Fc-fusion proteins consisting of protein antigen fused directly to IgG Fc fragment are transcytosed across respiratory mucosa by binding the neonatal Fc receptor (FcRn). **(C)** Protein antigens tethered to secretory IgA (sIgA) are transcytosed across respiratory mucosa by binding to Dectin-1 on the surface of M cells. **(D)** Protein antigens fused to C-terminal fragment of *Clostridium perfringens* enterotoxin (C-CPE) are transcytosed across respiratory mucosa by binding the tight junction protein Claudin-4 on the surface of M cells. **(E)** Polyethyleneimine (PEI) reversibly opens tight junctions and increases uptake of cyclodextrin-conjugated PEI mRNA polyplexes across the respiratory mucosa. **(F)** Inclusion of polyethylene glycol (PEG) into PACE-mRNA polyplexes increases mucus transport and therefore increases passive uptake across the respiratory mucosa. (Created with BioRender.com).

Protein-, peptide-, and polysaccharide-based subunit and non-viral mRNA vaccines are two strategies that address production, stability, and safety concerns of traditional live-attenuated and inactivated pathogenic vaccines. Yet mucosal subunit vaccines have historically been plagued by poor immunogenicity and failure to elicit long-lasting responses, due primarily to poor uptake across mucosal barriers. Several approaches have been employed to overcome these challenges in novel subunit and non-viral mRNA vaccines. To overcome lack of immunogenicity, subunit vaccines are typically administered with adjuvant to increase innate immune activation. Mucosal vaccine adjuvants have been reviewed elsewhere (155, 186). However, enhanced mucosal uptake should also be taken into account for mucosal adjuvants to facilitate spatiotemporal codelivery of antigen and adjuvant together for effective antigen presentation with costimulation. Here, we focus our discussion on approaches that facilitate enhanced uptake of vaccine components (and adjuvant where applicable) via active or passive transport across respiratory mucosal barriers, with a goal of delivering antigen and adjuvant to underlying MALT to prime robust mucosal immunity (**Figure 5**).

### Subunit vaccine approaches

#### Active transport via FcRn

The neonatal Fc receptor (FcRn) has been termed the ‘mucosal gateway’ for its potential to improve drug delivery across mucosal epithelial tissues in the nose, lungs, and gut (122, 153, 187, 188). Widely expressed on human mucosal epithelial tissues, FcRn serves to bidirectionally transcytose and recycle serum IgG and albumin (187). In fact, FcRn-expressing columnar epithelial cells are more abundant in the respiratory mucosa than M cells, making FcRn another attractive target for uptake (189). The structure, biology, and function of FcRn and its relevance to drug delivery are reviewed in depth by Pyzik et al. (190) and Sockolosky et al. (187). Researchers have harnessed FcRn’s role as a shuttle for IgG and albumin across mucosal epithelium to enhance the uptake of immune cargo, for example by making Fc-fusions between IgG Fc and a protein antigen subunit (191–194), or by modifying antigens with an albumin-binding lipid tail to ‘hitchhike’ on albumin across the mucosa via FcRn (**Figures 5A, B**) (4, 195). These strategies make use of the inherent function of FcRn to deliver subunit vaccine components across the mucosal barrier.

Rakhra et al. and Hartwell et al. pursued ‘albumin hitchhiking’ as a strategy to deliver peptide and protein antigens to the respiratory mucosa via albumin:FcRn-mediated uptake, which exploits albumin’s role as a fatty acid transporter (**Figure 5A**). Using intratracheal (i.t.) administration, Rakhra et al. demonstrated that modifying viral or cancer peptide T cell antigens and CpG adjuvant with an albumin-binding amphiphile tail enhanced uptake in the lungs and mediastinal LNs, leading to significantly greater Trm cells in the lung parenchyma compared to s.c. immunization with amphiphile vaccine or i.t. immunization with unmodified vaccine (195). Importantly, mice immunized i.t. with amphiphile-peptide and amphiphile-adjuvant vaccine exhibited 100% protection against i.t. viral challenge (195). Hartwell et al. developed intranasal vaccines against HIV and SARS-CoV-2 by modifying B cell protein antigens (engineered outer domain (eOD) gp120 env subunit of HIV and the SARS-CoV-2 RBD, respectively) with an albumin-binding amphiphile tail (4). Amphiphile protein conjugate vaccines exhibited increased uptake across nasal mucosal tissues in an FcRn-dependent manner, followed by FcRn-dependent expansion of GC B cells and Tfh cells in the NALT. Intranasal amphiphile protein vaccines generated 100- to 1000-fold higher antigen-specific IgG and IgA titers in serum and clinically relevant mucosal sites (respiratory mucosae and distal genitourinary mucosae) up to 34 weeks post prime compared to unmodified protein, including neutralizing antibodies against SARS-CoV-2 in the upper and lower respiratory mucosae, and long-lived IgA-secreting plasma cells in the genitourinary mucosa. Taken together, these data demonstrate that albumin-hitchhiking mucosal vaccines can harness FcRn to enhance uptake in both the upper and lower respiratory tracts to elicit robust resident B cell, T cell, and plasma cell responses in the mucosa.

Li et al. took a slightly different approach to FcRn targeting by fusing SARS-CoV-2 spike protein antigen directly to the IgG1 Fc fragment (S-Fc) (**Figure 5B**). Intranasal immunization with S-Fc plus adjuvant led to significantly higher IgG and IgA titers in serum, nasal washes, and bronchoalveolar lavage fluid 28 days post prime. Eight months post prime, mice immunized intranasally with S-Fc still showed 100-fold higher antibody-secreting cells in the bone marrow compared to mice immunized with unmodified spike protein (191). In a viral challenge study, Syrian hamsters immunized i.n. did not transmit virus to cohoused unimmunized hamsters and did not contract virus from cohoused infected unimmunized hamsters. In contrast, hamsters immunized i.m. both transmitted and contracted the virus when housed with unimmunized hamsters, albeit with lower viral titers than the unimmunized-to-unimmunized control groups (191). Other studies using the SARS-CoV-2 RBD (192), Herpes-simplex virus-2 glycoprotein D (193), and influenza hemagglutinin (194) demonstrated similar success with the IgG Fc fusion intranasal vaccine strategy. Using FcRn knockout models and mutant Fc fragments that cannot bind FcRn, it has been demonstrated that more robust immune responses following immunization with Fc fusion proteins can indeed be attributed to FcRn transcytosis as the knockout models and mutant proteins do not show the same enhanced immunogenicity (193, 194). These data suggest that intranasal vaccination utilizing the inherent function of FcRn to enhance uptake results in stronger functional immune protection at barrier sites.

#### Active transport via M cell targeting

Targeting intranasal vaccines to M cells is another strategy for active transport of vaccine antigen into MALT to generate robust mucosal immunity, particularly given the relative abundance and position of M cells in the FAE lining the SED of the NALT. In the gut, M cells in PPs bind and endocytose secretory IgA (SIgA), therefore coupling antigens to SIgA could serve to enhance intranasal uptake (**Figure 5C**) (196). Rochereau et al. coupled the HIV model antigen p24gag with secretory IgA (forming ‘p24-SIgA’) and showed that intranasal immunization with p24-SIgA plus adjuvant induced up to 100-fold higher serum and mucosal IgG and IgA titers in feces, vaginal lavage, and saliva when compared to unmodified p24 alone. Furthermore, p24-SIgA induced functional immune protection against p24-expressing vaccinia virus, as mice immunized with p24-SIgA showed complete survival following challenge (132).

M cells in the NALT express claudins on their surface that form tight junctions and seal off the intracellular space (197, 198). Targeting claudins could be another method to induce preferential sampling and active transport of vaccine antigens across mucosal epithelium. Using the C-terminal fragment of *Clostridium perfringens* enterotoxin (C-CPE), which is known to bind claudin-4, several groups have designed mucosal vaccines against diverse pathogens (**Figure 5D**) (108, 197, 199). Suzuki et al., for example, immunized mice intranasally with pneumococcal surface protein A (PspA) coupled to C-CPE (forming ‘PspA-C-CPE’), demonstrating with immunofluorescence that C-CPE vaccines bind M cells in the NALT (199). PspA-C-CPE intranasal immunization led to over 80% survival after respiratory challenge with S. pneumoniae, compared to vaccination with unmodified PspA and vehicle controls that led to only 60% and 15% survival, respectively (199). Intranasal immunization with influenza HA fused to CPE plus adjuvant induced 2- to 4- fold higher IgG and IgA titers in serum, bronchoalveolar lavage fluid, and fecal samples at 14 weeks post prime, suggesting the presence of resident plasma cells in lung and gut mucosae (108). Thus, targeting vaccine components to M cells by either fusing them to SIgA or C-CPE presents a promising strategy to increase transport across mucosal surfaces.

### ‘Pulling’ immune cells into the mucosa with prime-pull approaches

Following a parenteral intramuscular (i.m.) or subcutaneous (s.c.) prime with a mucosal boost, termed ‘prime and pull’ by Iwasaki et al, has shown success for generating protective mucosal and systemic immunity by ‘pulling’ circulating primed immune cells into mucosal tissues (200–204). ‘Prime-pull’ is capable of eliciting strong IgG and IgA in serum and mucosal fluids, such as bronchoalveolar lavage fluid and nasal wash following an intranasal pull (200, 202, 203), as well as resident CD4^+^ and CD8^+^ Trm cells (201–203). In contrast, i.m. prime and boost yields similar IgG responses but no detectable IgA or Trm cells (202). I.m. prime or i.n. boost alone yields significantly lower serum and mucosal antibody responses as well as fewer Trm cells in the lungs and nose when compared to i.m. prime plus i.n. pull (203). Haddadi et al. show that a mucosal pull with vaccine antigen is more effective for inducing CD8+ Trm cells when it is administered during the memory phase of the initial T cell response (201). Using the SARS-CoV-2 spike trimer, i.m. prime followed by i.n. pull led to complete survival following i.n. SARS-CoV-2 challenge, while i.m. prime alone resulted in less than 20% survival (203). Interestingly, when comparing i.n./i.n., i.m./i.n., and i.m./i.m. prime-boost dosing schemes, all led to different responses but resulted in complete survival following challenge with both Wuhan 1 and Delta strains of SARS-CoV-2 (202). Of note, prime-pull can also refer to i.n. prime followed by intravaginal administration of chemokines or adjuvants to ‘pull’ migrating immune cells into genitourinary tissues (205). Tregoning et al. demonstrated that immunizing mice intranasally with trimeric HIV gp140 followed by intravaginal pull with CCL28 or MPLA enhanced vaginal IgA titers, while s.c. prime followed by either MPLA or CCL28 intravaginal pull did not. In all, the preclinical evidence supporting ‘prime-pull’ as a strategy to promote mucosal immunity also highlights a clinical opportunity for mucosal vaccines to be used as a booster dose following previous prime or prime-boost series currently used in the clinic, for example to follow i.m. COVID vaccines to boost mucosal ‘hybrid immunity’ against SARS-CoV-2.

### Strategies for enhancing mucosal uptake of non-viral mRNA vaccines

mRNA vaccines provide an interesting alternative to the subunit vaccine strategies presented above, mainly for ease of production. mRNA vaccines allow for the production of difficult-to-manufacture protein complexes and structurally stable protein antigens (206). Several strategies have been explored to enhance diffusive uptake of mRNA vaccines across respiratory mucosal epithelium, such as using modified lipid nanoparticles (LNPs) to encapsulate mRNA (207) and incorporating polymers like polyethyleneimine (PEI) (208, 209) and polyethylene glycol (PEG) (210) into mRNA polyplexes.

LNPs are being investigated for mucosal targeted mRNA vaccines given the success of the parenteral intramuscular Pfizer (BNT162b2) and Moderna (mRNA-1273) SARS-CoV-2 mRNA vaccines that encoded the SARS-CoV-2 spike protein (211, 212). LNPs facilitate mRNA entry into cells but also possess inherent adjuvanticity when delivered parenterally and intranasally, allowing for codelivery of antigen and adjuvant in a costimulatory context required for immune activation (213, 214). Vaca et al. compared two different LNP formulations for intranasal delivery of mRNA (207). LNP1 was similar in formulation to the LNP used in Moderna’s mRNA-1273 vaccine, while LNP2 was modified to further optimize respiratory tract uptake with the inclusion of a cationic lipid. Using a Syrian hamster model and mRNA encoding a prefusion-stabilized SARS-CoV-2 spike protein, LNP2 exhibited a 10-fold dose enhancement over LNP1 when comparing serum IgG, IgA, and neutralizing antibody titers three weeks post boost (207). These data suggest that using cationic lipid-modified LNPs could be an effective strategy for enhancing mRNA vaccine uptake in the respiratory tract.

PEG and PEI polymers have also been used to increase uptake across the epithelial barriers of the respiratory tract (208–210). PEI crosses epithelial barriers by reversibly opening tight junctions, demonstrated by a decrease in the tight junction protein ZO-1 after treatment with complexes containing PEI (**Figure 5E**) (209). In addition, PEI exhibits inherent adjuvanticity (215), such that its incorporation into vaccine formulations can both enhance uptake across epithelial barriers and deliver antigen in a costimulatory context. Despite these advantages, PEI still poses safety concerns for respiratory delivery due to its high cationic charge (208). To combat this, Li et al. included cyclodextrin in PEI complexes to reduce the charge density while preserving the ability of PEI to condense nucleic acids (208, 209). Cyclodextrin conjugated with PEI2K (CP2K) demonstrated prolonged nasal residence time, upregulation of MHC I and costimulatory markers CD80 and CD86 on DCs, increased systemic and mucosal anti-HIV humoral responses, and antigen-specific CD8+ T cell lysis (209).

Lastly, PEGylation of nanoparticles has been shown to facilitate transport through mucus and thereby increases mucosal uptake (**Figure 5F**) (216). Using PEGylated poly(-amine-*co*-ester) (PACE) polyplexes, Suberi et al. demonstrated that intratracheal delivery of PACE-mRNA encoding the SARS-CoV-2 spike protein efficiently transfected epithelial and antigen presenting cells in the lung and BALF (210). 42 days post prime, mice immunized with PACE-mRNA exhibited significantly higher effector and memory lymphocytes, IgA class-switched B cells, and plasma cells compared to naive mice, as well as significantly higher survival rates following SARS-CoV-2 challenge (210). These data suggest that inclusion of PEI and PEG can increase transport of mRNA polyplexes across epithelial barriers to improve vaccination.

## Targeting the respiratory tract for tolerance

While mucosal delivery can be advantageous for eliciting protection against pathogens, it can also provide unique advantages as a target for inducing tolerance against autoimmunity. Development of new autoimmune therapies is urgently needed, as over 80 well-known autoimmune diseases impact approximately one in ten people worldwide with incidences increasing annually (217). Yet the most common practice for treating autoimmunity is with broadly immunosuppressive therapies such as monoclonal antibodies that inhibit or deplete effector immune cells (218, 219). While sometimes effective for treating disease, global immunosuppression renders patients susceptible to severe infections and cancer (16). An alternative approach, considered the “holy grail” of autoimmune therapy, is using ASIT to selectively target autoreactive cells in an antigen-specific manner (220). These therapies require identification of an appropriate autoantigen and delivery of this antigen as a peptide, protein, or host-expressed antigen via DNA/RNA in a ‘tolerogenic context’ – that is, in the absence of costimulation.

Peptide/protein antigens tend to be weakly immunogenic on their own, such that subunit vaccines often require an adjuvant to be administered with antigen to trigger an innate immune response that drives costimulatory signal expression for activation. Based on the two-signal model of lymphocyte activation, antigen presentation requires engagement of secondary costimulatory signals along with primary antigenic signal for antigen-specific immune activation (221, 222). Antigen presentation in the absence of costimulation leads instead to lymphocyte anergy, deletion, or differentiation into regulatory T/B cells – main mechanisms by which peripheral tolerance is maintained. In autoimmunity, on top of a loss of peripheral tolerance to autoantigen, higher baseline inflammation and inflammatory cytokines present from autoimmune damage can provide additional stimulatory context to propagate immune activation (223). To overcome this, administration of autoantigen *in the absence of adjuvant* via inherently tolerogenic mucosal routes may facilitate antigen presentation in a tolerogenic context that other administration routes may not provide (17, 224). Immune cells in mucosal barrier tissues and MALTs are predisposed to tolerance in order to maintain homeostasis against regularly encountered non-pathogenic antigens (17). For example, APCs underlying mucosal tissues express low levels of PRRs and TLRs, granting them a higher threshold for activation and a bias for tolerance so they actively induce regulatory T cells (Tregs) (140). Tregs play an essential role in maintaining peripheral tolerance through immune suppression in healthy individuals and restoration of self-tolerance in autoimmunity (225–227). Indeed, ‘oral tolerance’ (oral administration of antigen targeting the gut and GALT) has long been investigated as a strategy for restoring tolerance against autoantigens or allergens, given the gut’s ability to maintain homeostasis under high antigen exposure (228, 229). Some evidence suggests that nasal and pulmonary administration may outperform oral administration because the respiratory mucosa presents fewer barriers to uptake and allows for better bioavailability (230–232). Respiratory administration of ASITs can even induce generalized systemic tolerance, in some cases more effective than parenteral injections, and has been shown to prevent or ameliorate disease across multiple animal models of autoimmunity. The combination of these strategies highlights the promise of administering autoimmune therapies via the respiratory route to target NALT and BALT. Like mucosal vaccines, however, mucosal immunotherapies face challenges of poor uptake. Here, we present a rationale for pursuing respiratory delivery of tolerogenic immunotherapies and strategies for enhancing uptake, highlighting successful mechanisms of action and opportunities for further development.

### Intranasal route of administration for tolerance induction

The upper respiratory mucosa has been a target of immunomodulation through administration of cytokines, antibodies, and ASITs. Intranasal administration of cytokines IL-6 (233) and IL-10 (234) at high doses have been shown to induce tolerance in rat models of T cell mediated autoimmune disease, as evidenced by reductions in disease score, MHC-II expression on APCs, autoreactive lymphocyte proliferation, proinflammatory cytokines, and T cell and macrophage infiltrates – changes that were not observed with subcutaneous IL-10. Yet, the systemic nature of changes induced by cytokines given their complex interplay in the immune response limits the predictability and specificity of cytokine therapeutics. Antibodies face a similar challenge of broad immunomodulation even if localized delivery can be achieved with intranasal administration, for example, when targeting the CNS (235). Therefore, the rest of our discussion on intranasal tolerance will be focused on ASITs that selectively target autoimmune cells in an antigen-specific manner. Insights gained from studies on cytokine and/or antibody administration may still inform ASIT design, as in cases where anti-inflammatory cytokines are codelivered with antigen to enhance tolerance as shown by Li et al. (236).

Intranasal administration of peptide ASITs has yielded successful and lasting tolerance in mouse models of autoimmunity. O’Neil et al. prophylactically administered an antigen-specific peptide therapy intranasally in experimental autoimmune encephalomyelitis (EAE), a mouse model of multiple sclerosis (237), leading to reductions in disease score, histology scores, and protection against disease progression. Peptide-specific tolerance was also associated with reduced immune cell infiltration into the CNS and alleviation of disease in an IL-10-dependent manner. Regulatory CD4^+^ T_reg_ and B_reg_ cells are sources of IL-10 necessary for tolerance induction (225, 227, 238). O’Neil and others have shown that tolerance induction via peptides can also be affected by peptide solubility (239). These findings highlight the importance of formulation and encourage exploration of different immunotherapy delivery strategies.

Taking inspiration from vaccination strategies to enhance mucosal delivery may improve efficacy of tolerogenic ASITs as well, for example by targeting M cells. Cholera toxin (CT), while commonly recognized as a gold standard mucosal adjuvant in its native form, has evolved mechanisms that allow for uptake by mucosal M cells (240). When inactivated or neutralized by the SC, it can act as an inert mucosal delivery carrier (241, 242). Incorporation of inert, inactive, or non-immunogenic components of CT into fusion protein designs has shown promise for enhancing delivery and tolerogenic efficacy of peptide ASITs. Hansson et al. designed an antigen-specific fusion protein for intranasal delivery by combining an autoantigenic peptide with mutant enzymatically inactive cholera toxin A1 (CTA1)-subunit and a dimer of D-fragments from protein A (243). Similarly, Wang et al. developed a fusion protein termed CTB-GADIII, containing cholera toxin B subunit (CTB) fused to three tandem autoimmune peptides from glutamic acid decarboxylase 65 (GAD65), an autoantigen in the type-1 diabetes (T1D) mouse model (244). Intranasal administration of CTB-GADIII induced transcriptional changes in antigen presentation, cell signaling, and lipid metabolism in cDC1s to suggest a tolerogenic APC phenotype, leading to expansion of IL-10 secreting type 1 regulatory T (Tr1) cells and suppression of Th1 and Th17 autoimmune responses that were not observed with i.n. peptide or fusion protein alone. Furthermore, CTB fusion protein provided significant protection against T1D in NOD mice, while CTB alone did not. Together, these studies suggest that intranasal administration of disease-specific peptide(s) fused to an inactive CT subunit can enhance protection and amelioration of disease in mouse models of T1D, collagen-induced arthritis (CIA), experimental autoimmune myasthenia gravis (EAMG), and EAE compared to equimolar doses of peptide.

Administering more than one autoantigenic epitope, for example in the form of three peptides as Wang et al. investigated, is motivated by the phenomenon of ‘epitope spreading’ that is commonly observed in autoimmune pathogenesis. As an autoimmune disease progresses, autoantigen attack triggers damage and breakdown of surrounding tissues in chronically inflamed environments, leading to the spread of immune cell activation against other intramolecular and intermolecular epitopes (245–247). This poses a particular challenge for tolerance induction with antigen-specific immunotherapies that seek to induce tolerance against a single antigenic epitope. At certain stages of disease, if tolerance is induced to one antigenic epitope, an alternative epitope (either intermolecular or intramolecular) can even progress to become the main driver of the autoimmune response (247). While peptides inherently target only a single epitope, Metzler and Wraith suggested that enhancing a peptide’s affinity for MHC-II may afford broader epitope protection in autoimmune settings. They showed that intranasal administration of myelin basic protein (MBP) peptides with an amino acid mutation to enhance affinity led to significantly lower disease scores against both single epitope induced EAE *and* multi-epitope induced EAE compared to PBS control. Notably, oral administration of the same peptides did not significantly alleviate disease (230). Alternatively, delivering multiple epitopes simultaneously, either as multiple peptides or a full protein, could induce broader tolerance against autoimmune antigens that may be generated over the course of disease progression.

Accordingly, intranasal administration of soluble full proteins that contain multiple autoantigenic epitopes is an alternative strategy for inducing tolerance. This strategy has yielded promising results through suppression of Th1-mediated responses across multiple animal models of autoimmunity, including murine EAE using intranasal delivery of full MBP antigen (232), EAMG using nicotinic acetylcholine receptor (AChR) antigen (248), CIA using collagen II antigen, and the type 1 diabetes mouse model of non-obese diabetes (NOD) using GAD65 antigen (15). However, promising pre-clinical results have often not translated to success in the clinic. For example, when intranasal administration of a recombinant glycoprotein to treat rheumatoid arthritis was tested in a phase II clinical trial, intranasal administration of the full protein was found to be safe but not clinically effective (249). Lack of clinical efficacy in cases like this may derive from differences in mucosal uptake following intranasal administration in humans compared to small animal models. Dosing, frequency of administration, and resulting pharmacokinetics have a significant impact on mucosal tolerance induction following nasal administration (250). Differences in nasal anatomy and available surface area for uptake may play a role here, as the more complex turbinate structure in rodents provides a five-fold greater epithelial surface area to volume ratio compared to primates (159). Poor mucosal uptake may also have a more profound effect in humans and large animal models where a significantly larger dose is required compared to rodent models. These results suggest that more research into differences in respiratory delivery between small and large animal models and primates is warranted.

### Pulmonary route of administration for tolerance induction

Much like the upper airways of the nasal mucosa, the lower airways are a site of environmental antigen exposure and therefore a location of tightly regulated inflammation by regulatory immune cells, making pulmonary administration a promising route for autoimmune therapies. Pulmonary administration may also attain better bioavailability than oral administration by avoiding the harsh conditions of the gut, but with greater surface area than the nasal mucosa (231). Delivery to the lungs has been explored for tolerance induction based on the *‘hub-and-spoke’ hypothesis*, which postulates that lymphocytes traffic to the lung (*hub*) where they are licensed to effector sites (*spokes*) according to transcriptional changes in adhesion, activation, and locomotion molecules on their cell surface (160). Administering ASITs to the lungs could thus restore tolerance in autoimmune lymphocytes in an area they have to traffic through before entering effector sites to cause damage. Pulmonary administration is also attractive as a non-invasive option for delivery, especially for immunotherapies that may require frequent dosing. Recognizing that self-injections lead to poor patient compliance, Jin et al. pursued pulmonary administration of a peptide-based immunotherapy against MS and RA by investigating both instillation and insufflation methods of administration (161). Both methods yielded similar bioavailabilities (39.2 ± 5.2% and 44.5 ± 12.5%, respectively) and were considered safe and viable based on a lack of lung epithelial cell cytotoxicity. Aerosolization of autoantigens is another method for pulmonary delivery; administration of aerosolized autoantigenic proteins resulted in complete disease protection, decreased infiltrating inflammatory lymphocytes, and generation of lasting Tregs in EAE (232). Below we overview additional studies of pulmonary ASIT administration along with strategies to enhance their uptake and efficacy.

Carrier platforms can enhance efficacy of pulmonary administered autoantigens compared to unmodified peptides or proteins alone. Saito et al. investigated pulmonary delivery of antigen-specific peptide-containing particles for treatment of MS in murine EAE by testing two different sizes of particles with different trafficking properties: 15µm ‘micron-PLP particles’ that accumulated in the lung and 400-500 nm ‘nano-PLP particles’ that accumulated in the liver and spleen following i.v. administration (162). Repeated i.v. doses of lung-targeting micron-PLP particles induced tolerance while the same dose of nano-PLP did not. Nano-PLP that was administered intratracheally, however, yielded similar efficacy to i.v. micron-PLP. Of note, intratracheal nano-PLP led to an expansion of alveolar and interstitial macrophages and DC populations in the lung that expressed MHC-II but not CD86, suggesting reduced APC activation potential and a tolerogenic phenotype that could induce anergy upon antigen presentation to lymphocytes.

Using carrier platforms that enable multivalent display of autoantigens may further enhance tolerogenic antigen presentation through high avidity BCR binding in the absence of costimulation (163, 164). Thati et al. tested the therapeutic efficacy of a soluble multivalent peptide-polymer array in the EAE mouse model (165). Soluble antigen arrays (SAgAs), consisting of a hyaluronan polymer backbone co-grafted with autoantigenic peptide from myelin proteolipid protein (PLP) and intracellular adhesion molecule-1 (ICAM-1) ligand peptide (LABL, portion of LFA-1), was previously shown to induce tolerance in EAE when administered subcutaneously. Motivated by the hub-and-spoke hypothesis, Thati et al. then investigated pulmonary instillation compared to traditional subcutaneous and intravenous injection routes. Pulmonary instillation of SAgAs yielded clinical scores lower than both s.c. and i.v. administration. Kuehl et al. replicated the i.p. injection of SAgAs to evaluate cytokine and cellular responses compared to soluble PLP alone or PLP conjugated to LABL peptide via short linker (166). Pulmonary instillation with SAgA was most effective at reducing disease based on % weight loss, disease scores, and histological inflammation. Interestingly, splenocytes from SAgA-treated mice produced higher levels of IFN-γ, IL-6, and IL-17 compared to the PLP group. While classically considered inflammatory cytokines, IL-17, IL-6 and IFN-γ may also play anti-inflammatory roles by inhibiting IL-25 pathways in mucosal inflammation, inhibiting inflammatory cytokines while promoting IgA class switching in the context of slightly increased IL-10, and promoting tolerance of APC and regulatory T cell differentiation respectively (27, 251–253).

Due to the association of the BALT in autoimmunity, as previously discussed, it is unclear if pulmonary delivery of antigen in autoimmune settings with pre-established BALT will lead to BALT resolution or disease worsening. Thus, future studies should evaluate if trafficking to this tertiary lymphoid structure is helpful or harmful in patients with pulmonary autoimmune or allergic conditions.

### The role of IgA and sIgA in autoimmunity

**Figure 6:**
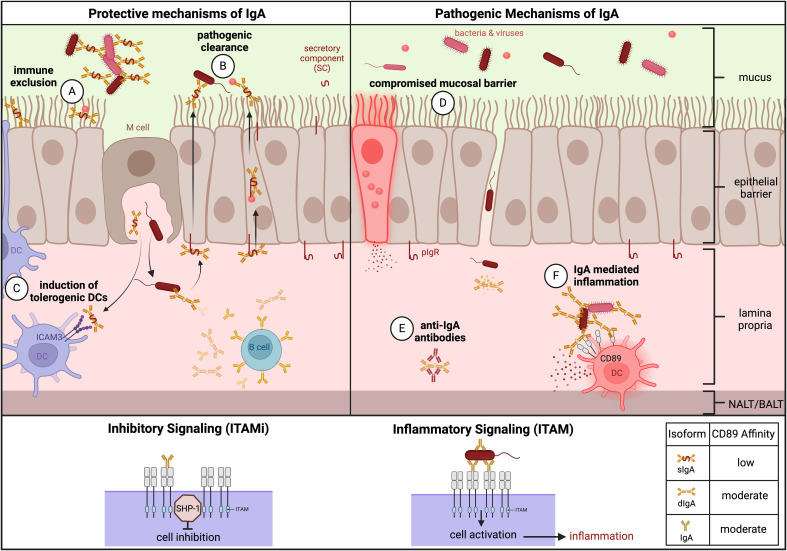
Protective and pathogenic mechanisms of IgA in autoimmunity. Protective – **(A)** Immune exclusion: IgA binds viral or bacterial pathogens in the mucosa, forming immune complexes to prevent pathogenic entry into or across the mucosal epithelium. **(B)** Pathogenic clearance: IgA clears pathogens that cross or infect the mucosal epithelium. **(C)** Induction of tolerogenic DCs: sIgA induces tolerogenic DCs via ICAM3 signaling. Pathogenic – **(D)** Compromised mucosal barrier: sIgA deficiency in combination with breaks in the mucosal barrier can allow for pathogen entry and infection. **(E)** Inflammation: IgA immune complexes in the lamina propria and serum can lead to crosslinking activation of CD89/FcαRi on monocytes and subsequent inflammation, contributing to autoimmune pathogenesis. **(F)** Anti-IgA autoantibodies: often IgG, can cause IgA deficiency. ITAMi and ITAM Signaling: Monomeric engagement of CD89 leads to ITAMi signaling and cell inhibition. Multivalent crosslinking of CD89 by IgA immune complexes with moderate avidity leads to ITAM signaling and cell activation. IgA affinity for CD89 varies based on isoform. (Created with BioRender.com).

**S**IgA production and secretion is a feature unique to the mucosal immune compartment. Thus, the role that IgA plays in tolerance induction following antigen administration to the respiratory mucosa may hold important insight to cellular mechanisms that drive autoimmune disease progression and amelioration. Here we overview IgA’s contributions to mechanisms of protection versus pathogenesis (**Figure 6**). Most of the studies discussed so far have not thoroughly investigated IgA responses to mucosal autoimmune therapies. Previous studies have shown that excess serum IgA or production of IgA autoantibodies contributes to chronic inflammation and tissue damage in many autoimmune diseases including rheumatoid arthritis, IgA nephropathy, IgA vasculitis, dermatitis herpetiformis, celiac disease, inflammatory bowel disease, Sjögren’s syndrome, ankylosing spondylitis, alcoholic liver cirrhosis, and acquired immunodeficiency syndrome (28). Conversely, sIgA deficiency and anti-IgA antibodies have also been reported to worsen many autoimmune diseases (254). To understand the complex role that IgA can play in autoimmunity and by extension autoimmune therapies, it is necessary to discern unique differences in IgA subclasses and IgA interactions with myeloid cells.

The only IgA receptor expressed by myeloid cells, FcαRI (CD89), is an immunoreceptor tyrosine-based activation motif (ITAM) receptor that grants IgA with dual functions that lead to either inhibition (via the ITAMi pathway) or activation (via the ITAM pathway) (28). Expression of the receptor on the cell surface is upregulated by N-formylmethionyl-leucyl-phenylalanine (fMLP), interleukin (IL)-8, TNF-α, lipopolysaccharide (LPS), and granulocyte-macrophage colony-stimulating factor (GM-CSF), and downregulated by TGF-β or interferon-γ (IFN-γ) (255). If CD89 is bound by monomeric IgA, an inhibitory pathway is induced via the ITAMi pathway (256). Alternatively, if bound by an immune complex, a pro-inflammatory immune response is induced by crosslinking via the ITAM pathway (**Figure 6**) (257).

A main protective mechanism of sIgA is preventing infection through immune exclusion by binding and capturing microbial antigen in the mucus to restrict uptake across the epithelium (**Figure 6A**) (258). dIgA plays a protective role in clearing microbes as well, by first binding to pathogen in lamina propria or epithelial cells while being transported by pIgR across the epithelium, where it is then cleaved on the luminal side to become sIgA with pathogen in tow (**Figure 6B**). Alternatively, dIgA can bind pathogen internally and form an immune complex that crosslinks CD89 on monocytes for immune activation and clearance. SIgA plays a more regulatory role in its interactions with CD89; it binds CD89 with a lower affinity than other IgA isoforms due to steric hindrance by the SC, is internalized by receptors that do not induce inflammatory DC maturation (256, 259), and instead induces regulatory DCs after binding to specific ICAM-3 grabbing non-integrin receptor 1 (**Figure 6C**) (260). DCs primed with sIgA (sIgA-DCs) are resistant to TLR-dependent maturation and induce Treg expansion via IL-10 (260, 261).

IgA mechanisms of pathogen clearance could ultimately play a role in maintaining immune tolerance as well as protection, as many autoimmune diseases are associated with molecular mimicry of bacteria or viruses that have breached the mucosal barrier (262, 263). A compromised mucosal barrier that allows bacterial overgrowth can initiate inflammation and activate MAIT cells, which then exacerbate inflammation and contribute to autoimmunity (**Figure 6D**) (264). Other pathogenic mechanisms related to IgA include presence of anti-IgA antibodies, shown to contribute to autoimmune pathogenesis (**Figure 6E**) (254). Altered glycosylation of IgA1, which is susceptible to bacterial proteases, can lead to conformational changes that increase the formation of immune complexes that are often linked to inflammation via activation of CD89 and IgA nephropathy (**Figure 6F**) (265). CD89 activation on immature DCs leads to MHC-II antigen presentation, maturation, production of inflammatory cytokines, and IL-10 secretion to promote IgA class switching in B cells (27).

Therefore, induction of sIgA in autoimmunity, especially in the context of mucosally-administered immunotherapies, begs further investigation. Elucidating the role of IgA in autoimmune pathogenesis and the effect of mucosal targeted immunotherapies on IgA may uncover important cellular and humoral mechanisms for inducing tolerance and would inform the design of future immunotherapies.

## Conclusions

In summary, mucosal lymphoid tissues of the upper and lower respiratory tract have demonstrated significant potential as a target both for immune activation and regulation. By leveraging our understanding of NALT and BALT biology, more effective strategies to activate and tolerize the immune system can be developed. The preclinical mucosal vaccines reviewed here focus on strategies to increase transport across epithelial barriers to underlying NALT/BALT, either through active or passive transport. Many of these studies show promising results with strong GC activation, enhanced systemic and mucosal humoral responses, and establishment of tissue-resident lymphocytes and/or plasma cells with enhanced vaccine uptake. Additionally, multiple groups have shown that antigen-specific immunotherapies delivered through the respiratory mucosa have the potential to be a prophylactic or therapeutic treatment method for mouse models of tissue-specific and systemic autoimmune diseases. While clinical data in humans is still limited, preclinical development of novel mucosal subunit vaccines and autoimmune ASITs that employ delivery mechanisms for enhanced mucosal uptake show promise for enhancing efficacy.

Going forward, continued development of novel mucosal vaccination strategies is still necessary to address an unmet need for safe and effective vaccines against mucosally-transmitted diseases, particularly for immunocompromised individuals who are not candidates for traditional live-attenuated pathogenic or vector-based vaccines. An emphasis should be placed on clinical translation, moving from preclinical small animal models into larger animal models and humans, as data here is limited especially with respect to uptake and trafficking behavior. Further investigation into the mechanism of lymphocyte recruitment to mucosal inductive sites as well as subsequent activation and dissemination to effector sites following vaccination would help guide vaccine development as well. Lastly, gaining a more complete understanding of sIgA generation, its role in regulation, and other immune mechanisms that follow mucosal administration of antigen in different contexts would aid the development of safer and more effective autoimmune therapies.
